# Populations of the Invasive Mussel *Mytella strigata* in China Showed Lower Genetic Diversity in Autumn than in Spring

**DOI:** 10.3390/biology14010016

**Published:** 2024-12-27

**Authors:** Peizhen Ma, Chenxia Zuo, Shaojing Yan, Xiangyu Wu, Xiaojie Ma, Yi Zhu, Zhen Zhang

**Affiliations:** 1 Laboratory of Marine Organism Taxonomy & Phylogeny, Qingdao Key Laboratory of Marine Biodiversity and Conservation, Institute of Oceanology, Chinese Academy of Sciences, Qingdao 266071, China; mapz@ysfri.ac.cn (P.M.);; 2 State Key Laboratory of Mariculture Biobreeding and Sustainable Goods, Yellow Sea Fisheries Research Institute, Chinese Academy of Fishery Sciences, Qingdao 266071, China; 3 Laboratory for Marine Fisheries Science and Food Production Processes, Qingdao Marine Science and Technology Center, Qingdao 266237, China; 4 College of Life Sciences, Qingdao University, Qingdao 266071, China; 5 Hainan Provincial Key Laboratory of Tropical Maricultural Technology, Hainan Academy of Ocean and Fisheries Sciences, Haikou 571126, China; 6 University of Chinese Academy of Sciences, Beijing 100049, China

**Keywords:** biodiversity, biological invasion, mussel, population genetics

## Abstract

Native to tropical America, the charru mussel, *Mytella strigata*, has been spreading rapidly in the West Pacific Ocean, including the South China Sea. In order to study the adaptive evolution of *M. strigata* and examine the present status of invasion in China, the mitochondrial *nad2* gene fragment was employed to analyze the genetic variations of seven populations sampled in both spring and autumn 2023. Results showed that all the populations had high haplotype diversity (>0.5) and low nucleotide diversity (<0.005), suggesting the ongoing rapid expansion following a genetic bottleneck. The Zhanjiang population had the highest genetic diversity in spring with 22 haplotypes, 37 polymorphic sites, and haplotype diversity, nucleotide diversity, and the average number of nucleotide differences being 0.911, 0.00623, and 4.341, respectively. However, in autumn, the Shanwei population had the most haplotypes (11) and polymorphic sites (19), with the highest haplotype diversity value of 0.891, while the Qunjian population had the highest nucleotide diversity (0.00392) and average number of nucleotide differences (2.809). Combining geographic populations by seasons confirmed lower genetic diversity in autumn compared to spring, evidenced by fewer haplotypes and polymorphic sites, reduced haplotype diversity and nucleotide diversity, and lower genetic distance within populations. These findings provided evidence for understanding the molecular characteristics of *M. strigata* population expansion in China.

## Simple Summary

The invasive charru mussel, *Mytella strigata*, has been spreading rapidly in the West Pacific, including the South China Sea. This study analyzed genetic variations in seven populations sampled in both spring and autumn 2023 using mitochondrial *nad2* gene fragments. Results revealed high haplotype diversity but low nucleotide diversity for all populations, suggesting recent rapid expansion after a genetic bottleneck. On the whole, genetic diversity was higher in spring populations compared to autumn populations. These findings highlight seasonal differences in genetic variation and provide insights into the molecular characteristics of *M. strigata* population expansion in China.

## 1. Introduction

Biodiversity has been declining in recent years [1], with climate change and biological invasions as two causes [2]. Biological invasions are a partial cause of recent species extinctions, with climate change exacerbating these impacts [3,4,5]. Human activities, especially global maritime trade, contribute to aquatic bioinvasions [6]. Ballast water and hull fouling serve as two primary pathways for the long-distance spread of alien aquatic species [7,8]. Additionally, ocean and coastal currents facilitate the spread of invasive species across small areas [9]. Mollusks and crustaceans have the highest number of marine invasive species [10]. In particular, many mussels are successful invasive species, facilitated by their fast growth, early reproductive maturity, high fecundity, strong adhesion capabilities, and adaptability to diverse habitats [11,12,13,14].

*Mytella strigata* (Hanley, 1843), also called the charru mussel, is native to the Pacific and Atlantic coasts of tropical America [15]. It first invaded Florida in the 1980s and subsequently spread to the western Indo-Pacific region, including the Philippines [16], Singapore [15], Thailand [17], India [18], and China [19,20,21,22]. *Mytella strigata* can tolerate a wide range of salinities (2–40) [23] and inhabits diverse environments such as mud flats, lines, shells of farmed oysters, and plastic film covering float rafts [16,19]. In addition, this species has high reproductive capacity, with single female individual producing over 1 million eggs per year [24]. These characteristics make *Mytella strigata* a potent global invader. *Mytella strigata* causes substantive losses to the aquaculture industry [25,26,27,28,29,30] and reduces native biodiversity in the invaded regions [30,31]. In China, *Mytella strigata* has spread to Taiwan, Hong Kong, Hainan, Guangdong, Guangxi, and Fujian [19,20,21,22] and may pose a threat to mariculture and local biodiversity [32,33].

Genetic variation is essential in understanding the success of biological invasions [34]. A positive correlation exists between the probability of invasion success and genetic diversity in the founding population [35,36]. However, genetic bottlenecks during species invasion can reduce genetic variation [36,37,38]. The bottleneck effect and subsequent inbreeding further limit the adaptive potential of invasive individuals [39,40]. In addition, population genetic studies are crucial for identifying invasion sources, assessing the status of invasive populations, monitoring their spread, and understanding genetic variation and molecular evolution [41]. Both within- and between-population variation are crucial for long-term evolutionary fitness and short-term individual adaptability [42]. Genetic markers, such as the rapidly evolving mitochondrial cytochrome oxidase subunit I (*COI*), are commonly used in the classification, phylogeography, and population genetics of metazoans [43,44]. Studies on *COI* of *Mytella strigata* supported that Colombia would be the source of the *Mytella strigata* populations in China, and Chinese populations had just experienced or were experiencing a population bottleneck [19,21,45]. However, the presence of doubly uniparental inheritance (DUI) and heteroplasmy in *COI* sequences, namely matrilineal lineage (*F-COI*) and patrilineal lineage (*M-COI*), poses challenges in genetic analysis due to the divergence between the two types [19,45,46]. Due to its faster evolutionary rate and greater genetic variation, the *nad2* gene was identified as a more suitable marker for population genetic studies of *Mytella strigata*. Population genetic analysis using this gene suggested that *Mytella strigata* populations in China might have passed through a bottleneck period and are currently in a state of population expansion [21].

The rapid spread of *Mytella strigata* in China has created an urgent need to monitor its population genetic dynamics. This study conducted genetic analysis on *Mytella strigata* from different populations and seasons. Our aims were to: (1) compare the genetic diversity of *Mytella strigata* populations from time and spatial scales; (2) explore the factors influencing the population genetics of *Mytella strigata*; (3) identify the current invasion status of *Mytella strigata* in China.

## 2. Materials and Methods

### 2.1. Sample Collection

**Figure 1:**
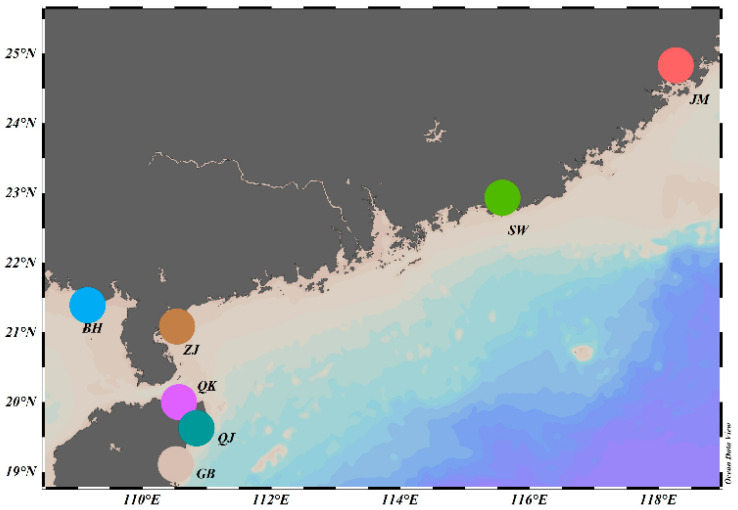
Chinese populations of *Mytella strigata* sampled in this study.

A total of 508 *Mytella strigata* individuals from seven populations in intertidal areas in China were collected in 2023, including Jimei (JM), Shanwei (SW), Zhanjiang (ZJ), Beihai (BH), Qukou (QK), Gangbei (GB), and Qunjian (QJ) (Figure 1, Table 1). 286 individuals were collected in spring, and 222 were collected in autumn from the same locations. These specimens were preserved in 95% alcohol and preserved in the Laboratory of Marine Organism Taxonomy & Phylogeny, Qingdao Key Laboratory of Marine Biodiversity and Conservation, Institute of Oceanology, Chinese Academy of Sciences.

### 2.2. DNA Extraction and Sequencing

The genomic DNA of each mussel was extracted using the Tiangen DNA kits (DP324, Tiangen Biotech (Beijing) Co., Ltd., Beijing, China) following the instructions strictly, and only the adductor muscles were used. Then the partial *nad2* gene was amplified with primers developed by Yan et al. (2023). A 25 μL PCR amplification system was conducted in this study, which was made up of 12.5 μL of 2 × Taq PCR MasterMix (PC1120; Beijing Solarbio Science & Technology Co., Ltd., Beijing, China), 10.5 μL of ddH_2_O, 1.0 μL of template DNA, and 0.5 μL of each primer. The PCR procedure involved initial denaturation at 95 °C for 3 min; 32 cycles of DNA denaturation (95 °C for 30 s), temperature reduction (48 °C for 1 min), and polymerase (72 °C for 1 min); and a final extension at 72 °C for 5 min. After detecting the purity by agarose gel electrophoresis, the PCR products were sequenced using an Applied Biosystems 3730xl Genetic Analyzer (Beijing Tsingke Biotech Co., Ltd., Beijing, China).

### 2.3. Data Analysis

Sequence lengths were optimized after alignment and minimal manual trimming to retain maximum genetic information. Five parameters, including the number of haplotypes (*h*), number of polymorphic (segregating) sites (*S*), haplotype (gene) diversity (*Hd*), nucleotide diversity (*Pi*), and the average number of nucleotide differences (*K*), were analyzed in this study using DnaSP v6.12.03 software to evaluate the diversity of *Mytella strigata* populations both in spring and autumn [47]. MEGA v7.0.26 software was used for sequence alignment and to assess genetic distances of *Mytella strigata* populations in spring and autumn, respectively [48]. PopART v1.7 and Arlequin v3.5 were used to construct haplotype networks [49,50]. Arlequin v3.5 was also utilized to perform the molecular variance (AMOVA) of the populations. The genetic differentiation coefficient (F-statistics, *Fst*) of inter-population was calculated using Arlequin v3.5 [50]. To examine seasonal genetic variation with larger datasets, all geographic populations from the same season were combined. Genetic diversity parameters (*h*, *S*, *Hd*, *Pi*, and *K*) of spring populations and autumn populations and their haplotype relationships were analyzed using the methods mentioned above.

## 3. Results

### 3.1. Genetic Diversity Analysis Within Spring and Autumn Populations

**Figure 2:**
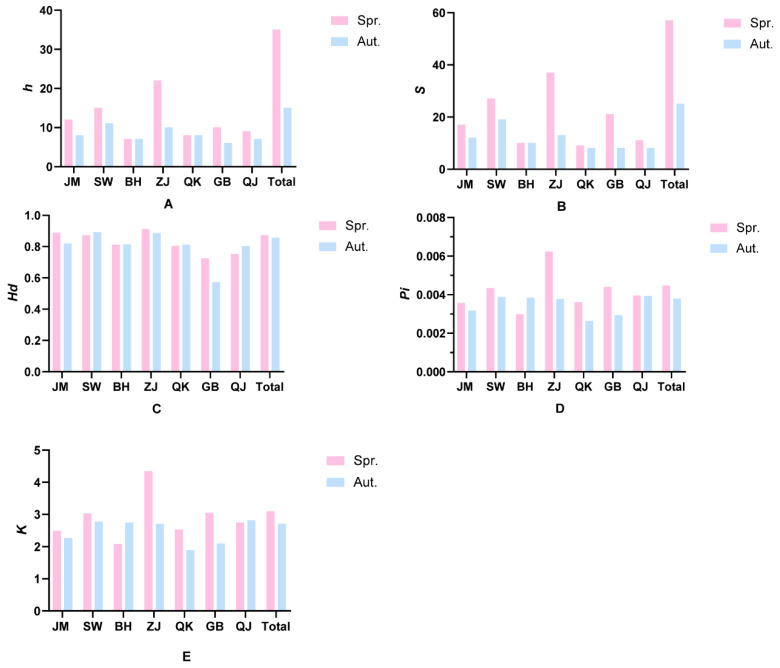
The bar chart of genetic diversity parameters for spring and autumn populations of *Mytella strigata*. (**A**–**E**) represent the differences in *h*, *S*, *Hd*, *Pi*, and *K* parameters between the spring and autumn populations of *Mytella strigata*, respectively.

*Nad2* sequences of all 508 individuals were successfully obtained, with 286 from spring (GenBank accession Nos. PQ560842-PQ560876) and 222 (GenBank accession Nos. PQ553550-PQ553564) from autumn populations. In the spring populations, the sequences were optimized to the length of 699 bp. ZJ population showed the highest values of *h* (22), *S* (37), *Hd* (0.911), *Pi* (0.00623), and *K* (4.341), while the BH population had the lowest values for *h* (7), *Pi* (0.00297), and *K* (2.077). The QK population had the lowest value of *S* (9), and GB had the lowest *Hd* (0.725). For the autumn populations, the effective sequence length was 716 bp. The SW population had the highest values of *h* (11), *S* (19), and *Hd* (0.891), *K* (2.770), and the QJ population had the highest value of *Pi* (0.00392). The GB population showed the lowest values of *h* (6), *S* (8), and *Hd* (0.572), and the QK population had the lowest values of *Pi* (0.00263) and *K* (1.886). The total values of *h* (35), *S* (57), *Hd* (0.872), *Pi* (0.00446), and *K* (3.088) were higher in the spring populations than the autumn populations with *h* (15), *S* (25), *Hd* (0.855), *Pi* (0.00378), and *K* (2.703) (Figure 2). In BH, both populations had equal *h* and *S*, but the autumn population displayed greater *Hd*, *Pi*, and *K* than the spring population. Apart from the ZJ spring population, the *Hd* values in both spring and autumn populations in other regions were greater than 0.05, and the *Pi* values were less than 0.005.

### 3.2. Genetic Distance, Genetic Differentiation Coefficient, and AMOVA Analysis

Genetic distances between spring populations ranged from 0.00330 (BH-JM) to 0.00613 (ZJ-GB). The lowest genetic distance within spring populations was observed in BH (0.00299), and the highest genetic distance within populations was observed in ZJ (0.00639) (Table 2). In autumn populations, genetic distances between populations ranged from 0.00288 (JM-QK) to 0.00480 (SW-GB), with QK and QJ having the lowest (0.00265) and highest (0.00396) intra-population distances, respectively (Table 3). Genetic distances within autumn populations were smaller than those in spring, with reductions ranging from 0.00039 to 0.00259, except in the BH and QJ populations, which showed slight increases of 0.00087 and 0.00001, respectively. Genetic distances among the BH-JM, BH-SW, BH-GB, and BH-QJ populations were higher in autumn than in spring, increasing by 0.00005 to 0.00034. In contrast, genetic distances in the remaining autumn populations were lower than in spring, reducing from 0.00004 to 0.00197.

Genetic differentiation coefficients of QJ-SW decreased, while they increased for the GB-JM, GB-SW, and GB-QK in autumn compared to spring. AMOVA analysis of the spring populations showed that 7.40% of the genetic variation occurred between populations and 92.60% within populations, whereas the autumn populations showed 10.44% of the genetic variation between populations and 89.56% within populations (Table 4). AMOVA results revealed an increase in variation (3.04% higher) between populations and a decrease (3.04% lower) in variation within populations in autumn compared to spring.

### 3.3. Genetic Relationships of Haplotypes

**Figure 3:**
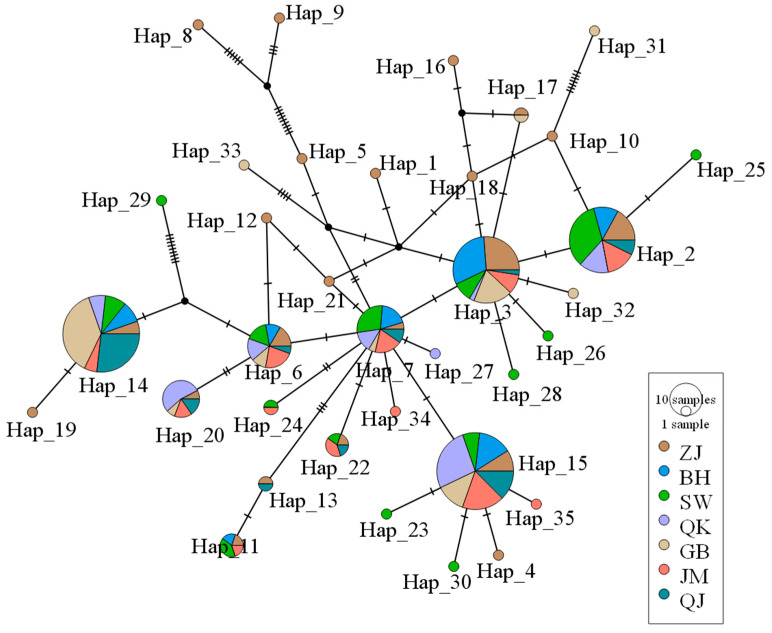
A TCS Network of *Mytella strigata* populations collected in spring 2023.

**Figure 4:**
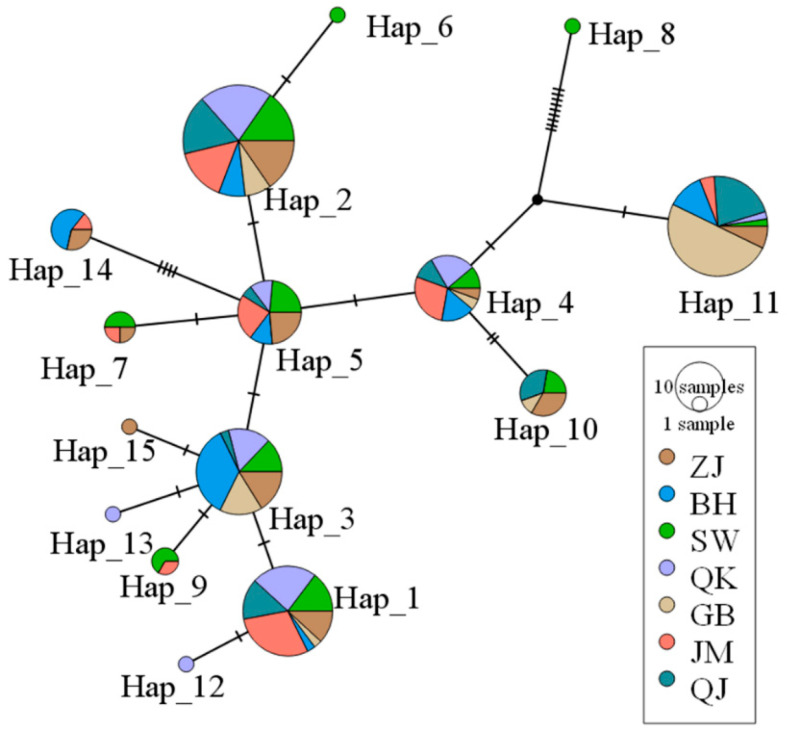
A TCS Network of *Mytella strigata* populations collected in autumn 2023.

The haplotype network map of the spring populations was a “star-shaped” distribution, centered on Hap_7 (Figure 3). Among the 35 haplotypes, Hap_14 (56 sequences), Hap_15 (56 sequences), and Hap_3 (42 sequences) were the dominant haplotypes. The seven spring populations shared Hap_3, Hap_6, Hap_7, Hap_14, and Hap_15. There were 11 unique haplotypes in the ZJ population, whereas there were no unique haplotypes in the BH population among spring populations. A total of 15 haplotypes were found in autumn populations, and the stellate distribution was not obvious (Figure 4). Hap_1 (34 sequences), Hap_2 (52 sequences), and Hap_11 (42 sequences) were the dominant haplotypes. The SW and QK autumn populations had two unique haplotypes: Hap_6 and Hap_8, Hap_12 and Hap_13, respectively. ZJ autumn population had one unique haplotype (Hap_15). Overall, the number of haplotypes decreased in the autumn populations compared to the spring populations.

### 3.4. Genetic Diversity Analysis and Genetic Relationships of Haplotypes Between Spring and Autumn Populations

**Figure 5:**
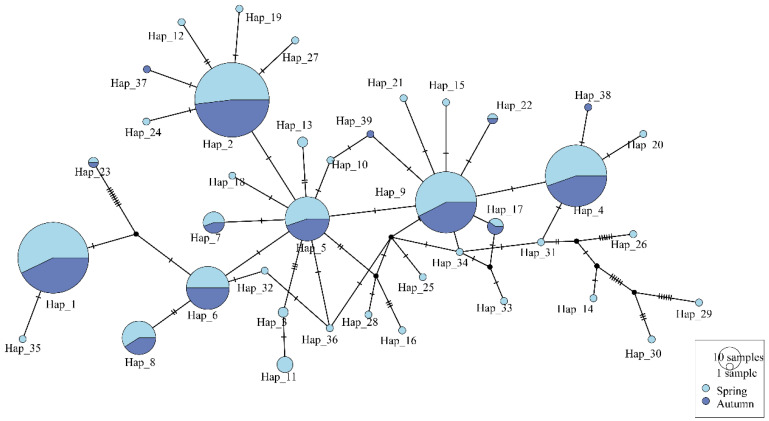
A TCS Network of spring and autumn populations of *Mytella strigata*.

508 sequences (699 bp) were used for genetic diversity and genetic relationships of haplotypes analysis. The values of *h*, *S*, *Hd*, *Pi*, and *K* were lower in the autumn populations compared to the spring populations (Table 5). The *Hd* values for both spring (0.873) and autumn (0.857) populations exceeded 0.05. The *Pi* values of spring (0.00454) and autumn (0.00391) were less than 0.005. The dominant haplotypes included Hap_1 (98 sequences), Hap_2 (108 sequences), Hap_4 (74 sequences), and Hap_9 (73 sequences) (Figure 5). The spring population exhibited 24 unique haplotypes, while the autumn population had only 3.

## 4. Discussion

The genetic variations of seven *Mytella strigata* populations sampled in spring and autumn in intertidal areas in China indicated lower population genetic diversity in autumn, evidenced by fewer haplotypes and polymorphic sites, reduced haplotype diversity and nucleotide diversity, and lower genetic distance within populations. One possible explanation was that the summer heat influenced the survival and genetic variation in *Mytella strigata*. Intertidal animals suffer from temperature variations, especially across seasons [51,52]. Some animals would not survive the hottest summer temperatures because of physiological damages [53]. Han et al. (2020) found the nucleotide diversity of *Mytilisepta virgata* (Wiegmann, 1837) collected in different seasons showed significant differences, with the lowest values observed in summer, possibly due to the death of some genotypically heat-sensitive individuals [54]. Increased seawater temperatures in the summer might lead to oxidative stress in marine bivalves, and heat stress could lead to mitochondrial failure, which affected shellfish health and population size [55]. DNA damage of *Ruditapes decussatus* (Linnaeus, 1758) and *Mytilus galloprovincialis* Lamarck, 1819 was also seasonally related and significantly higher in summer [56]. As an intertidal species, the optimal survival temperature for *Mytella strigata* ranges from 20 to 23 °C [57]. With increasing intensity and duration of heat stress, *Mytella strigata* showed significant decreases in respiration rate [58]. Mortality rates increased significantly under heat stress, reaching approximately 70% after 30 days at 28 °C and 80% after 30 days at 31 °C [57]. In this study, the average temperatures at sampling sites were as follows: 22.9–24.5 °C during spring (March–May), 28.3–29.5 °C during summer (June–August), and 26.2–27.0 °C during autumn (September–November), according to World Sea Temperatures (https://www.seatemperature.org/, accessed on 11 December 2024). As a result, *Mytella strigata* populations in China are likely to be exposed to heat stress during the summer, which might be an important factor for the decreased genetic diversity. Additionally, the *nad2* gene mutation rate per generation must be considered, as the spring and autumn samples might represent different generations [59].

*Fst* is widely used as an index of population structure [58]. Low to moderate genetic differentiation (*Fst* < 0.15, *p* < 0.05) was observed in spring populations, while great genetic differentiation (*Fst* > 0.25, *p* < 0.05) occurred in autumn populations, particularly for GB-JM (0.30308), GB-SW (0.28486), and GB-QK (0.36668). Both *Hd* and *Pi* were lower in the autumn population compared to spring. However, the values of *Hd* were greater than 0.05 for both the spring and autumn populations, and *Pi* values were less than 0.005 for all populations except the spring ZJ population. These high *Hd* and low *Pi* values suggested that the Chinese *Mytella strigata* populations were in a period of rapid expansion after the bottleneck effect [21,60]. Bottleneck effects experienced by invasive alien species may result in significant genetic differentiation among invasive populations, as evidenced by variation between populations increasing and variation within populations decreasing in autumn compared to spring in this study. Goodisman et al. (2001) suggested that frequent bottlenecks experienced by the introduced wasp *Vespula germanica* populations in Australia led to the observed varied genetic patterns, as well as a genetic pattern in which the genetic distance between invasive populations was positively correlated with geographical distance [61].

## 5. Conclusions

In the present study, the mitochondrial *nad2* gene fragment was employed to analyze the genetic variations of seven *Mytella strigata* populations sampled in spring and autumn 2023. All populations exhibited high haplotype diversity (>0.5) and low nucleotide diversity (<0.005), suggesting the ongoing rapid expansion following a genetic bottleneck. However, population differentiation of *Mytella strigata* gradually formed. Overall, genetic variation was lower in autumn populations, evidenced by fewer haplotypes and polymorphic sites, reduced haplotype diversity and nucleotide diversity, and lower genetic distance within populations. Our study was limited to analyzing seasonal variations between spring and autumn using a single genetic marker (*nad2*). The application of multiple genetic markers or combinations of different types of genetic markers could potentially influence the observed results of genetic variation. In addition, more evidence is needed to elucidate the factors contributing to the reduction in genetic variation in *Mytella strigata*, as observed in autumn compared to spring in this study.

## Figures and Tables

**Table 1:** Information on the samples collected and the number of sequences obtained.

	Spring Populations		Autumn Populations	
Location	Collection Date	Quantity	Collection Date	Quantity
JM	6 April 2023	39	9 November 2023	32
SW	5 April 2023	46	8 November 2023	32
BH	3 April 2023	38	13 November 2023	30
ZJ	2 April 2023	46	16 November 2023	32
QK	1 April 2023	40	10 November 2023	33
GB	1 April 2023	44	11 November 2023	33
QJ	1 April 2023	33	11 November 2023	30
Total	-	286	-	222

**Table 2:** Genetic distance (*GD*) (diagonal and below diagonal, shown in orange) and genetic differentiation coefficient (F-statistics, *Fst*) (above diagonal, shown in blue) between spring populations of *Mytella strigata*.

	JM	SW	BH	ZJ	QK	GB	QJ	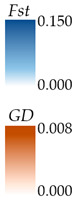
JM	0.00358	0.01769	0.00554	0.02433 *	0.00027	0.12765 *	0.11658 *	
SW	0.00405	0.00437	–0.00330	0.00800	0.06691 *	0.13440 *	0.13736 *	
BH	0.00330	0.00367	0.00299	0.00187	0.05826 *	0.12876 *	0.14584 *	
ZJ	0.00512	0.00543	0.00471	0.00639	0.06309 *	0.12230 *	0.13411 *	
QK	0.00360	0.00429	0.00351	0.00535	0.00363	0.12044 *	0.09469 *	
GB	0.00458	0.00507	0.00425	0.00613	0.00457	0.00442	–0.01128	
QJ	0.00426	0.00483	0.00405	0.00598	0.00418	0.00414	0.00395	

* indicates significant level *p* < 0.05.

**Table 3:** Genetic distance (diagonal and below diagonal) and genetic differentiation coefficient (F-statistics, *Fst*) (above diagonal) between autumn populations of *Mytella strigata*.

	JM	SW	BH	ZJ	QK	GB	QJ	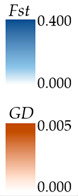
JM	0.00319	–0.01121	0.03176	–0.00411	–0.01404 *	0.30308 *	0.05834	
SW	0.00351	0.00392	0.02438	–0.01320	–0.00418	0.28486 *	0.05571 *	
BH	0.00364	0.00399	0.00386	–0.00696	0.06174 *	0.21617	0.04579	
ZJ	0.00348	0.00381	0.00381	0.00380	0.01717	0.25530	0.03219	
QK	0.00288	0.00327	0.00347	0.00328	0.00265	0.36668 *	0.10397	
GB	0.00441	0.00480	0.00435	0.00454	0.00443	0.00292	0.09723 *	
QJ	0.00379	0.00417	0.00410	0.00401	0.00368	0.00383	0.00396	

* indicates significant level *p* < 0.05.

**Table 4:** AMOVA results of *Mytella strigata* populations. collected in spring 2023. Values before “/” represent spring populations, and values after “/” represent autumn populations. *d.f.*: degrees of freedom.

Source of Variation	*d.f.*	Sum of Squares	Variance Components	Percentage of Variation
Among populations	6/6	36.902/34.605	0.11540/0.14317 Va	7.40/10.44
Within populations	279/215	403.108/246.035	1.44483/1.22807 Vb	92.60/89.56

**Table 5:** Genetic diversity parameters of spring and autumn populations of *Mytella strigata*.

	*h*	*S*	*Hd*	*Pi*	*K*
Spring	36	57	0.873	0.00454	3.127
Autumn	16	26	0.857	0.00391	2.720
Total	39	58	0.864	0.00427	2.939

## Data Availability

Relevant information has been added in the article.
